# Determination of velocity correction factors for real-time air velocity monitoring in underground mines

**DOI:** 10.1007/s40789-017-0184-z

**Published:** 2017-11-16

**Authors:** Lihong Zhou, Liming Yuan, Rick Thomas, Anthony Iannacchione

**Affiliations:** 1 Pittsburgh Mining Research Division, Office of Mine Safety and Health Research, National Institute for Occupational Safety and Health, PO Box 18070, 626 Cochrans Mill Road, Pittsburgh, PA 15236, USA; 2 University of Pittsburgh, Pittsburgh, PA, USA

**Keywords:** Underground mine, Airflow measurement, Correction factor

## Abstract

When there are installations of air velocity sensors in the mining industry for real-time airflow monitoring, a problem exists with how the monitored air velocity at a fixed location corresponds to the average air velocity, which is used to determine the volume flow rate of air in an entry with the cross-sectional area. Correction factors have been practically employed to convert a measured centerline air velocity to the average air velocity. However, studies on the recommended correction factors of the sensor-measured air velocity to the average air velocity at cross sections are still lacking. A comprehensive airflow measurement was made at the Safety Research Coal Mine, Bruceton, PA, using three measuring methods including single-point reading, moving traverse, and fixed-point traverse. The air velocity distribution at each measuring station was analyzed using an air velocity contour map generated with Surfer^®^. The correction factors at each measuring station for both the centerline and the sensor location were calculated and are discussed.

## 1 Introduction

As the lifeblood of a mine, ventilation not only provides fresh air for personnel to breathe but also dilutes and carries away toxic or explosive gases and dust. Ensuring an efficient mine ventilation system is critical to the health and safety of underground mine workers. In the history of mining, many fatal mine gas and dust explosions have been attributed to inadequate mine ventilation. The primary objective of ventilation is to control air distribution underground to satisfy statutory and safety requirements for air quantity and quality. The airflow rate is a key ventilation design parameter in managing and improving the effectiveness of mine ventilation.

With the steadily increasing atmospheric monitoring systems (AMS) installations in underground coal mines in the U.S. for the detection of fire in the belt entry, the real-time monitoring of airflow velocity in some critical locations has attracted attention (Martikainen et al. 2010; Belle 2013). Based on Rowland et al.’s (2017) survey of 235 U.S. underground coal mines, 21 of the mines are using air velocity sensors. Outside of the U.S., such as United Kingdom, Canada, South Africa and Poland, real-time ventilation monitoring in mines is a leading practice in the mining industry. Especially in South Africa, almost all collieries have been using real-time air velocity monitoring devices (Belle 2013).

The benefits of real-time airflow monitoring in underground mines are many. Monitors provide:

1. Early warning of a weakening trend in airflow or a ventilation failure,
2. Evaluate the performance of the underground environmental conditions with long-term real-time monitoring airflow data, and
3. Help mine operators and emergency response personnel decide how to respond to underground emergency.

However, most of the real-time airflow sensors obtain a fixed-point air velocity at designated installation locations. The fixed-point air velocities need to be converted to average volume airflow rates to be used in ventilation design and ventilation performance evaluations. Traditionally, the determination of air volume flow rate is achieved by using a vane anemometer or smoke tube in conjunction with an area measurement. Conversely, the constraints imposed on the fixed-point sensors are more rigorous because they are capable of measuring the airflow at only one point (Thimons and Kohler 1985).

With the application of fixed-point air velocity sensors for the AMS, the question arises as to how the readings from these sensors can be related to average air volume flow rate. The concept of a “correction factor” has been used to convert the velocity at the centerline to the average velocity, which is required to compute average volume flow rate (Thimons and Kohler 1985). Substantial work has been performed by numerous investigators to address the correction factor issue. Despite this, there are no universally accepted practices, or even agreement about the proper correction factors among practicing engineers (Kohler and English 1983; Thimons and Kohler 1985). For example, a correction factor of 0.9 is recommended by Kingery (1960) to covert the centerline velocity to the average velocity, while 0.8 is recommended by McPherson (2009). Hartman et al. (1997) also recommended 0.8 based on the relation of velocity ratio of average velocity to centerline velocity and Reynolds number. An extensive study on correction factors was completed by Kohler and English (1983), including a thorough literature search and extensive in-mine experiments. They found that the correction factors published in the literature, as well as that obtained from their in-mine experiments, span a larger range and are often contradictory. It was concluded that although the reported correction factors in the literature were widely varying and sometimes contradictory, they were most likely appropriate and representative of the mines in which the experiments were conducted. The generation of correction factors is therefore encumbered by their site-specific nature, which is not easily quantifiable.

The Safety Research Coal Mine (SRCM), Bruceton, PA, at the Pittsburgh Mining Research Division (PMRD) of the National Institute for Occupational Safety and Health (NIOSH) has an AMS equipped with eight monitoring stations to collect real-time underground environment data for research purposes, including air velocity, carbon monoxide, temperature, oxygen, etc. The fixed-point air velocity monitoring data from the airflow sensors are frequently required to be converted to average volume airflow rate to analyze and evaluate the ventilation performance. Using correction factors to convert the fixed-point air velocity to the average air velocity is thought to be a very fast and convenient method. However, all the correction factors obtained in previous research refer to the conversion from the centerline velocity to the average velocity. Air velocity sensors may be mounted below the roof and at the centerline to avoid interfering with mine operations.

Based on the authors’ best knowledge, no sensor location correction factor has been studied and published so far. To address this need, an extensive air velocity study was conducted at the SRCM to obtain the site-specific correction factors for the current and potential future sensor locations in the mine. The objective of this paper is to establish a set of correction factors for the selected locations at the SRCM.

## 2 Airflow survey

The SRCM is a full-scale underground mine facility located at NIOSH’s Bruceton Campus in Pittsburgh. It is a room-and-pillar mine approximately the size of a working section with one intake entry and one return entry. A Joy Axivane Series fan is installed at the surface above the return shaft to exhaust air from the mine. In the mine, a Conspec^®^ AMS is equipped at eight monitoring stations to monitor mine environment conditions such as barometric pressure, temperature, airflow velocity, humidity, and oxygen, and to detect fire by way of carbon monoxide, carbon dioxide, and smoke sensors. The locations of monitoring stations are frequently changed depending on the requirements of the experiments conducted in the mine. The air velocity sensors are mounted about 0.3 m (1 ft) below the roof at each monitoring station.

**Fig. 1:**
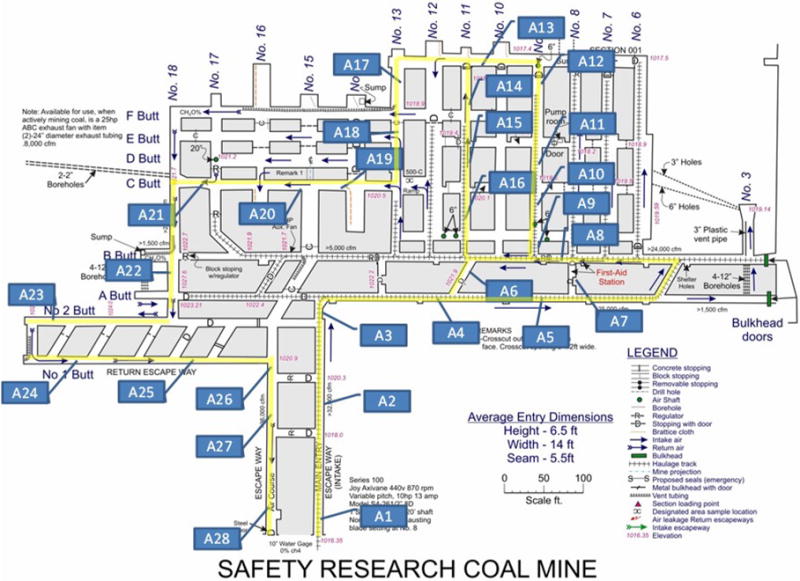
SRCM layout and airflow measuring stations

**Fig. 2:**
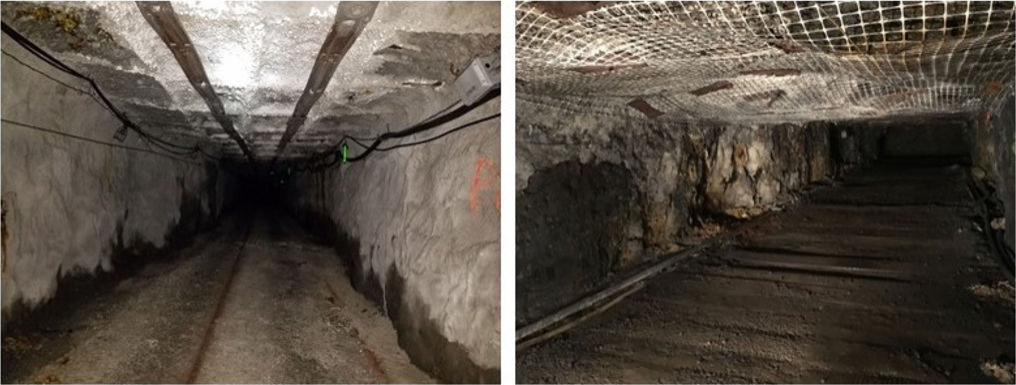
Entries of measuring location A4 (left) and A 22 (right)

A thorough study of the ventilation was conducted in the SRCM to obtain the average air velocities, air velocity at the centerline, and at 0.3 m (1 ft) below the roof at 28 airflow measuring locations. The established 28 airflow measuring locations and the SRCM layout can be seen in Fig. 1. Most of the airways at SRCM are rectangular. Figure 2 shows a picture of entries where measuring location A4 and A22 locate.

Calibrated vane anemometers were used for measurements at all stations. The anemometers were attached to a 1.5-m rod to reduce the turbulence effect due to the presence of the observers. The time duration for all the measurements was set to 1 min. The three most commonly used air velocity methods—single-point readings, moving traverses, and fixed-point traverses—were used in the air velocity survey, as described below.

**Fig. 3:**
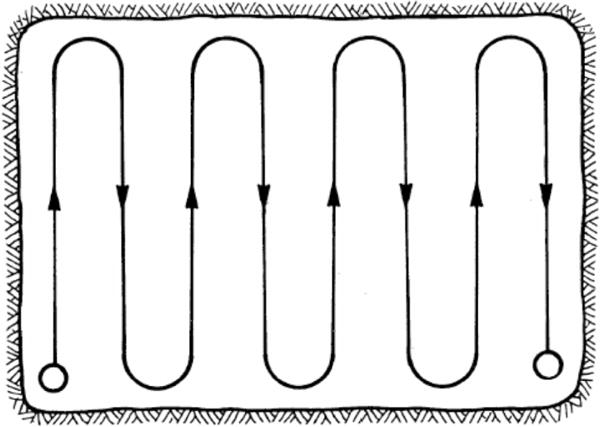
Path of a moving anemometer traverse (McPherson 2009)

**Fig. 4:**
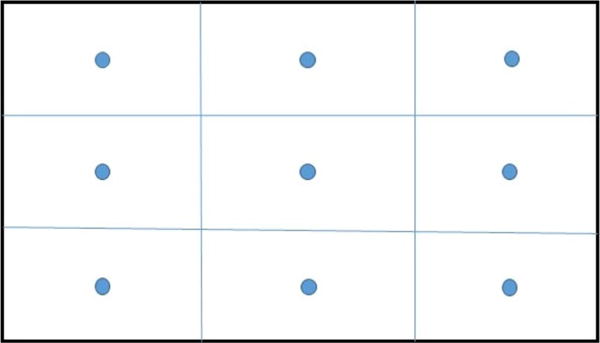
Measuring spots of the fixed-point traverse method

1. Single-point reading: The single-point readings were made at the centerline of the section and 0.3 m (1 ft) below the roof at each measuring station. The single-point reading is thought to be the most common but least accurate method among the three methods, as it does not necessarily represent the average air velocity. In reality, the readings from the AMS air velocity sensors are all single-point readings.
2. Moving (or continuous) traverse: The anemometer is slowly moved through a plane at right angles to the axis of the airway to take moving traverse readings. The moving traverses are most frequently used to improve the measurement accuracy. The time interval for conducting measurements varies with the accuracy desired. Figure 3 shows a typical path of a moving anemometer traverse used in this survey.
3. Fixed-point traverse: Spot measurements at a number of locations are taken over the cross section. The cross section of airway may be divided into subsections of equal area, and the individual measurement is taken in the center of each subsection. In the airflow survey, the cross section was divided into nine subsections with the measuring spot located at the center of each subsection (as shown in Fig. 4). One of the benefits of the fixed-point traverse is the ease of obtaining the distribution of air velocity in a plane.

It was found that the fixed-point traverse measurement at the airflow measuring stations from A8–A12 sometimes received “questionable” readings because of flow reversal and pulsing. In the context of the mine-wide airflow distribution, these airflow measuring stations were located in the lowest airflow zones and were eliminated from further exploration and analysis to avoid ambiguity. In addition, the two moving traverse readings at airflow measuring station A14 were removed due to significant deviations. Therefore, the readings from the rest of the 21 measuring locations, including the moving traverse, the fixed-point traverse, and the centerline velocity, are processed and analyzed in this paper.

## 3 Correction factors

The correction factor, Ccenterline, from the centerline air velocity to the average air velocity is defined as

(1) $$C_{\mathrm{centerline}}=\frac{V_{\mathrm{average}}}{V_{\mathrm{centerline}}}$$

where, *V_average_* is the average velocity at a cross section and *V_centerline_* is the fixed-point air velocity at the centerline of the cross section.

Similarly, the correction factor from the sensor location air velocity, C_sensor_, to the average air velocity can be written as

(2) $$C_{\mathrm{sensor}}=\frac{V_{\mathrm{average}}}{V_{\mathrm{sensor}}}$$

where, *V_sensor_* is the fixed-point air velocity at the sensor location.

At each measuring station, the air velocity at the centerline and at the sensor location were evaluated and the average air velocity calculated. The centerline correction factors and sensor location correction factors can be obtained from the measured data using Eqs. (1) and (2).

### 3.1 Measured average air velocity

The average air velocity at each measuring station needed to be determined to calculate the correction factors. This can be directly measured using the moving traverse method or calculated from the nine fixed-point velocity readings of the fixed-point traverse method. The moving traverse method is the most frequently used technique to measure the average air velocity at a cross section. This method saves time when measuring average air velocity comparing to the fixed-point traverse, but it cannot yield the distribution of the air velocity. This can only be accomplished with the fixed-point traverse method. If only the average velocity is needed, the moving transverse method is the first choice.

**Fig. 5:**
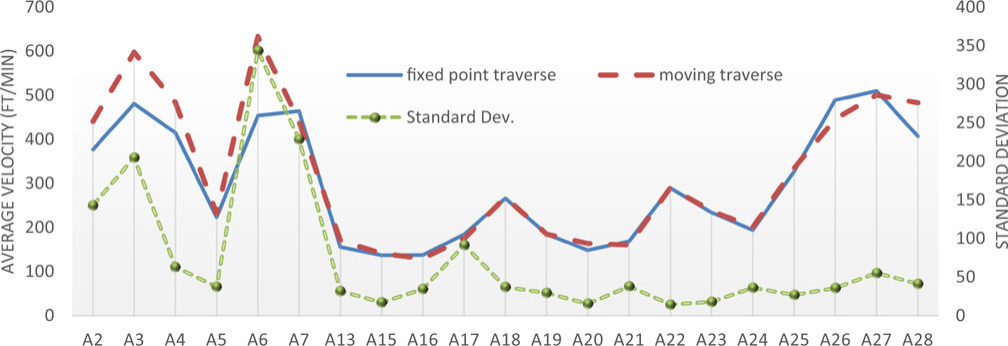
Measured average air velocities from the fixed-point traverse and the moving traverse, and the standard deviation of the measured air velocities from the fixed-point traverse method

Figure 5 shows the comparisons of the average air velocities measured from the fixed-point traverse and the moving traverse. The average air velocities from the moving traverse method were directly measured in the survey, whereas the average air velocity from the fixed-point traverse method was calculated from the nine fixed-point velocity readings at each cross section using the grid-based mapping program. Surfer^®^ applies interpolation methods to calculate the unknown air velocities of desired points by referring to the measured air velocities of neighboring points. Among the 12 interpolation methods offered by Surfer^®^, Kriging was selected as the method to produce an accurate grid of the air velocity measurement data. Kriging is a geostatistical gridding method producing visual maps from irregularly spaced data that has proven to be useful and popular in many fields (Golden Software, Inc. 2012). Kriging attempts to express trends suggested in the data and it incorporates anisotropy and underlying trends in an efficient and natural manner (Yang et al. 2004). Surfer^®^ was also used in this study to visualize the air velocity distribution at each cross section, which will be discussed later in the paper.

It can be seen from Fig. 4 that the moving traverse method appears to obtain higher readings than the fixed-point traverse method at four measuring stations. The measured average air velocities from the two methods match very well, especially in the range of 0.5–1.5 m/s (100–300 fpm). However, the differences in the average velocities of the two measuring methods at locations A2, A3, A4, and A6 exceed 0.3 ms/s (60 ft/min), where the air velocities exceed 2.0 m/s (400 fpm). It is premature to say whether the greater velocity causes the larger difference between the moving traverse method and the fixed-point traverse method. As can be seen in Fig. 5, the average air velocities from fixed point traverse and moving traverse are very close at both measuring locations A26 and A27, although both the measured air velocities are above 2.0 m/s (400 fpm). The magnitude of air velocity may not be a contributor to the differences observed between these two measuring methods.

Kohler and English (1983) noted that errors might be caused while moving the anemometer from an area of lower to higher velocity or from higher to lower velocity. With further analysis of the data from A2, A3, A4, and A6, the point velocities at the nine measurement grid locations in the fixed-point traverse have a very wide range of values. Taking an example of locations A3 and A6, which has the largest differences in average velocities from the two methods, the nine measured grid air velocities range from 0.7 m/s (132 fpm) to 4.7 (923 fpm) and from 0.8 to 3.3 m/s (168–652 fpm), respectively. In contrast, the air velocity distribution was more uniform at A26 and A27, with the nine measured grid velocities falling into relatively narrow ranges of 2.1–2.6 m/s (406–516 fpm) and 2.0–2.9 m/s (402–567 fpm), respectively.

From the above analysis, it can be concluded that the wider range of air velocities at these measuring locations might have contributed to the large deviation of the measured average velocities from the two methods. To investigate the correlation between the air velocity distribution and the deviation of the two air velocity measuring methods, the standard deviation at each measuring station was calculated using the nine measured fixed-point velocities. As can be seen in Fig. 5, the larger the standard deviation at a measuring location, the greater the differences between the air velocities using the two measuring methods. With the current data, it is difficult to say which method produced more accurate measurements. To minimize the error in the calculation of the correction factors, the air velocities from the two methods at each measuring station were averaged and used for the correction factors analysis.

### 3.2 Calculation of correction factors

**Fig. 6:**
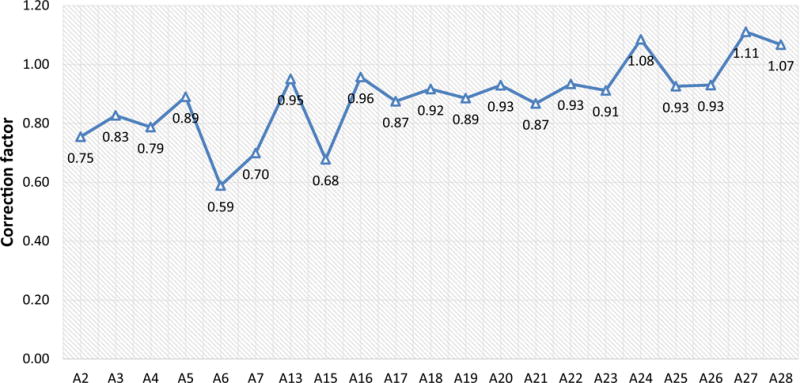
Correction factors of the centerline air velocities to the average velocity

At each measuring station, two correction factors—the centerline correction factors and sensor location correction factors—were calculated using Eqs. (1) and (2). The centerline correction factors have been extensively reported from 0.6 to 1.1 (Kohler and English 1983). All the correction factors of the centerline air velocities to the average velocities for the 21 measuring locations were calculated using the ratios of the measured average air velocity to the measured centerline single-point air velocity (as shown in Fig. 6). It can be seen that most of the correction factors fall into the range of 0.8–1.0, with an overall average of 0.88 and a standard deviation of 0.13. The 95% confidence interval is 0.82–0.94, which means there is a 95% possibility in the SRCM that the centerline correction factor falls into the range of 0.82–0.94. As the maximum air velocity is mostly at the centerline in a typical duct flow, most of the centerline correction factors are below 1.0. As can be seen in Fig. 6, there are only three correction factors above 1.0, which were at measuring locations A24, A 27, and A28. The measuring station A6 had the smallest correction factor, 0.59.

**Fig. 7:**
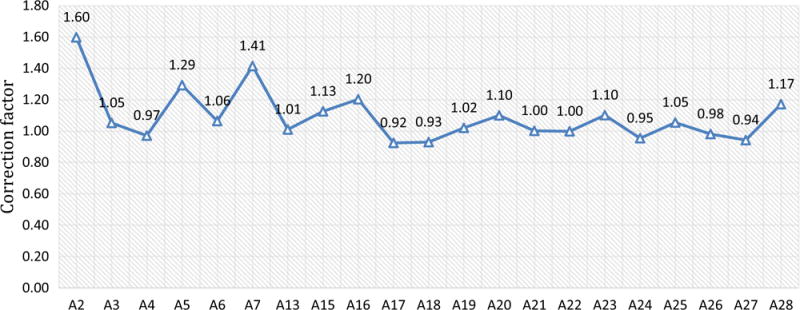
Correction factors of the air velocities at the sensor locations to the average air velocity

The sensor location correction factor is defined as the ratio of the average air velocity to the fixed-point velocity at the sensor location which is at center and 0.3 m (1-ft) foot below the roof. The sensor location correction factors for all 21 measuring locations range from 0.92 to 1.60 with an average of 1.09 and a standard deviation of 0.17 (as shown in Fig. 7). Unlike the centerline correction factors, the majority of the correction factors at the sensor locations are larger than 1. This indicates that the air velocities at most sensor locations [0.3 m (1 ft) below the roof] are smaller than the average air velocity. If the air velocity sensor readings are directly used to calculate the average airflow rate with the cross section areas, the airflow rates will be underestimated in most cases.

**Fig. 8:**
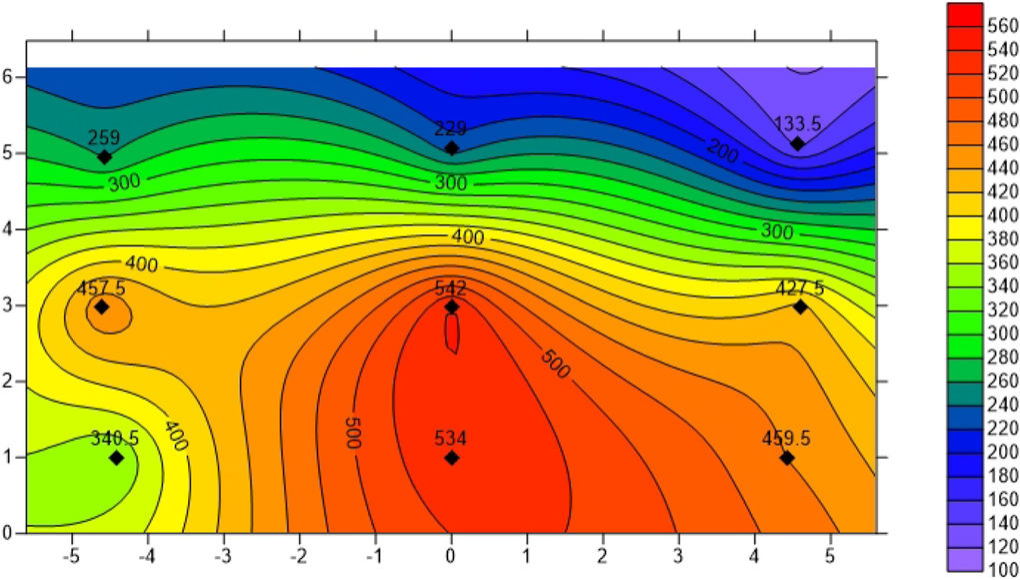
Air velocity (fpm) contour map at A2. Unit of physical dimension is in ft

The largest sensor location correction factor was obtained at the measuring location A2, and about 50% of the correction factors were above the average correction factor of 1.09. To determine the cause of the large correction factor at measuring location A2, the air velocity contour map (shown in Fig. 8) was generated using Surfer® with the 10 fixed-point measurements, including 9 from the fixed-point traverses and 1 from the centerline. From the contour map generated for the measuring location A2, the larger air velocity zone was located at the center and bottom part of the rectangular cross section. The near-roof zone where the sensor was mounted had the smaller air velocity. Therefore, the air velocity at the sensor location was much smaller than the average air velocity, and this yielded a much larger correction factor. The formation of the air velocity distribution at A2 will be discussed later in Sect. 4.

At some point, we can say that the correction factor at a site is largely determined by the air velocity distribution. The value of each sensor location correction factor obtained in this paper, either larger than 1 or smaller than 1, can be well explained using the generated velocity contour map at each measuring station.

As mentioned earlier, the average sensor location correction factors obtained from 21 measuring locations at the SRCM is 1.09. A question then arises as to whether the application of an overall correction factor to all the locations can yield satisfactory results. Taking the value of 1.09 as the overall sensor location correction factor at the SRCM, the error caused in the calculated average velocity compared to the measured average velocity at each measuring location is calculated and listed in Table 1. The positive values indicate that the application of the overall correction factor overestimates the average air velocity. With the average value of 1.09 as the overall sensor location correction factor, at 9 out of 21 measuring locations, the value causes more than 10% error in the calculated average air velocities.

Another way to investigate whether the application of overall sensor location correction yields any improvements in the determination of the average air velocity is to compare the error using the average sensor correction factor and the error caused by reading the sensor directly as the average air velocity without correction. The latter, named as error without correction, is calculated using Eq. (3) and displayed in Table 1 for each measuring location. The maximum error using the point sensor reading directly as the average air velocity exists at the measuring location A2 with the error of 37%.

(3) $$\mathrm{Error}=\frac{V_{\mathrm{sensor}}-V_{\mathrm{average}}}{V_{\mathrm{average}}}\times \;100\%$$

**Fig. 9:**
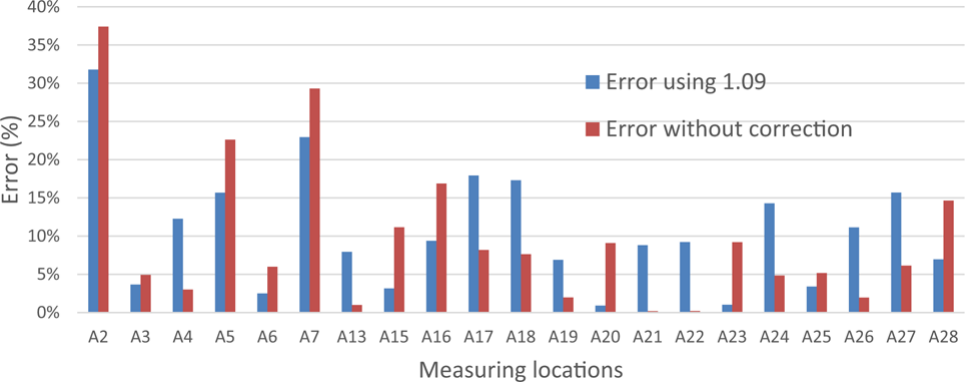
Comparison of absolute values of error using average correction factor and without correction

Figure 9 presents the absolute values of the error using 1.09 as the overall correction factor and the error without correction at each measuring location. It can be seen that the use of the overall correction only improves the accuracy in the calculation of the average air velocity at 10 of 21 measuring locations. At the rest of the locations, the use of the overall correction factor actually make the case even worse. This finding suggests that applying an overall sensor location correction factor to convert a point sensor reading to the average air velocity is of little help in improving the accuracy of the average air velocity. These results show that using the sensor location correction factor measured specifically at each measuring location can obtain a better outcome.

## 4 Sensor location and the velocity profile

In this study, the sensor location correction factors were obtained based on the assumption that the sensors are at the center and 0.3 m (1 ft) beneath the roof. In mining practice, sensors do not have to be mounted in this manner. Although there is no regulation about specifications for air velocity sensor locations, carbon monoxide or smoke sensors are required to be installed near the center in the upper third of the entry, in a location that does not expose personnel working on the system to unsafe conditions (30 CFR 75.351). To avoid interference with moving equipment and miners, air velocity sensors might be installed at the corner close to the roof and ribs. Any change to the sensor location will result in a change to the sensor reading, and thus the correction factor.

Zhou et al. (2012) investigated the impact of an obstacle on the fixed-point sensor velocity readings using a well-calibrated computational fluid dynamics model. It was found out that the mounted location of a velocity sensor plays an important role in the sensor readings at a cross section, due to the irregular velocity distribution at the plane where the sensor is mounted. It is well known that when air flows through a duct, whether rectangular or circulation, the duct does not have a uniform velocity over the cross section of the flowing air, but will have a “velocity profile.” When air enters a mine entry, the air particles in the layer in contact with the surface of the entry come to a complete stop because of the “no-slip” condition. This layer also causes the air particles in the adjacent layers to slow down gradually as a result of friction. To make up for this velocity reduction, the velocity of the air at the mid-section of the entry has to increase to keep the mass flow rate through the entry constant. As a result, a velocity gradient develops along the entry. Ideally, the maximum velocity should occur at the centerline of the duct and the minimum should occur near the wall. However, this is not likely the case for underground mine airways, due to extreme roughness of the airways and the merging and splitting of air paths.

**Fig. 10:**
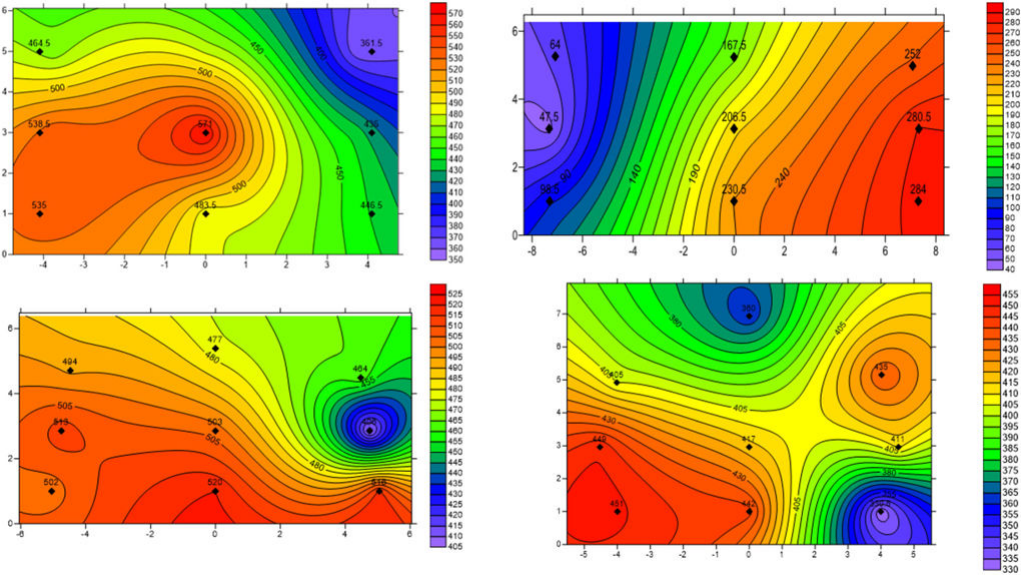
Selected air velocity (fpm) contours at measuring stations A4, A17, A26, and A27. Unit of physical dimension is in ft

To investigate the actual air velocity profile and its impact on the value of correction factors at each measuring location, a velocity contour map was generated at each measuring plane using measured fixed-point traverse air velocities. The fixed-point traverse method may not be used very often within the mining industry because the time consumed for one section measurement may exceed 30 min, depending on the number of the measuring points. However, one of the advantages of applying this method is to be able to generate the distribution of the airflow at the measuring sections. Figure 10 displays four typical contour maps at the selected measuring planes A4, A17, A26, and A27 plus A2 shown in Fig. 8 for the purpose of illustration. The rest of contour maps were not presented in the paper to conserve space. As can be seen from Fig. 10, none of the velocity distributions follow the ideal velocity profile with the velocity gradually decreasing from the center to the perimeter of the plane, and the same is true for the contour maps not displayed in this paper. The obstructions in the entry, changes in entry cross section, rapid floor elevation changes, and bends and turns in air flow were responsible for the diverse contour plots.

The maximum air velocity at measuring location A4 occurs from the center to the left bottom corner, and the lowest air velocity is at the right upper corner. At measuring location A17, the air velocity decreases from the lower right corner to the upper left corner. The maximum air velocities are observed near the entry floors for measuring locations A26 and A27. A sensor location correction factor is determined by comparing the air velocity and the measured average air velocity. The velocity distribution at a plane does not affect the average velocity significantly, but it does affect the sensor readings. Apparently, a sensor mounted in a high-velocity zone will yield a smaller correction factor than one mounted in a low-velocity zone. Using measuring location A17 as an example, a larger correction factor will be obtained if the sensor is at the left side than that at the right side, as the air velocity exhibits an increasing trend from left to right.

With a known velocity distribution, it is straightforward to explain the reading from the sensor. However, the velocity distributions are often not available. A known well-established turbulent velocity profile can only be obtained in a sufficiently long (usually 30 times the diameter) airway with smooth walls (Care et al. 2013), which is rare in mining practice with bends, intersections, raises, and equipment.

In this study, no two velocity distributions are the same among the total 21 velocity contour maps. With careful observation and analysis, it is found that the velocity distribution at a measuring plane is mainly determined by the measuring locations. For example, the measuring location A2 is located in the main intake about 30 m to the mouth of the portal, which is not sufficiently long to allow the airstream to become a fully developed flow. As can be seen in Fig. 8, the air velocity at the bottom section is greater than that at upper section. The air passing through A4 is split into two streams, with one stream flowing straightly forward and the other portion of air taking a route on the left to the working section. Therefore, the airflow at the measuring location A4 “shifts” to the left side, with the consequence that the left side of the section has a slightly higher air velocity than the right side. For the air velocity distributions at A26 and A 27, which are located in the main return, the large air leakage from the main intake through the bottom of the door merges into the main air stream, which causes the maximum air velocity to occur near the bottom of the floors.

The other contour maps also reveal a similar relationship between the location and the air velocity distribution pattern. These results indicate that a certain air velocity distribution pattern is well correlated with the location of the measuring station, mainly affected by the merging and splitting of the airflow upstream and downstream.

## 5 Conclusions

1. A comprehensive airflow study using single-point reading, moving traverse, and fixed-point traverse methods was conducted at the Safety Research Coal Mine, Bruceton, PA, to determine the appropriate conversion factors for sensor-measured air velocities. Measurement results demonstrate that differences in the measured average air velocities between the moving traverse method and the fixed-point traverse method exist at a cross section where the air velocity spans a wide range. The two methods generate close average velocities when the air velocity variations are in a small range. With the current data, it is difficult to say which method produces more accurate measurements. More research may be needed for future work.
2. The velocity contours indicate that the air velocity distributions at most measuring locations do not follow the typical “velocity profile” observed within a duct, where the maximum velocity occurs at the centerline of the duct and the minimum occurs near the wall. A certain air velocity distribution pattern is correlated well with the location of the measuring station, mainly because of the merging and splitting of the airflow upstream and downstream.
3. Both the centerline correction factors and the sensor location correction factors were determined based on the measurement data, and proved to be site-specific. There is no general correction factor that applies to all the cases. The sensor location correction factors for the 21 measuring locations varied from 0.92 to 1.60. A majority of sensor location correction factors obtained in this paper are larger than 1.00.
4. The sensor location correction factor is mainly determined by the air velocity distribution at the measuring plane. Using a single correction factor for all the locations will produce certain errors in the calculation of the average air velocity from the sensor readings. An effective method for obtaining the appropriate sensor location correction factor is to measure the air velocity at the sensor location. Then determine the average air velocity to get the correction factor for the specific site before a sensor is mounted. However, airflow merging and splitting can affect the air velocity distribution significantly, and the measurements need to be made to update the correction factor if any air course changes occur upstream or downstream.

## Figures and Tables

**Table 1:** Errors in the calculated average velocity with and without the overall correction factor

Measure locations	Width × height (ft)	Sensor correction factor	Error using 1.09 (%)	Error without correction (%)
A2	11 × 6	1.60	−32	−37
A3	12 × 6	1.05	4	−5
A4	9 × 6	0.97	12	3
A5	9 × 7	1.29	−16	−23
A6	5 × 7	1.06	2	−6
A7	4 × 7	1.41	−23	−29
A13	14 × 6	1.01	8	−1
A15	12 × 6	1.13	−3	−11
A16	13 × 7	1.20	−9	−17
A17	16 × 6	0.92	18	8
A18	17 × 6	0.93	17	8
A19	17 × 6	1.02	7	−2
A20	16 × 6	1.10	−1	−9
A21	12 × 7	1.00	9	0
A22	12 × 5	1.00	9	0
A23	12 × 5	1.10	−1	−9
A24	14 × 6	0.95	14	5
A25	11 × 5	1.05	3	−5
A26	11 × 6	0.98	11	2
A27	11 × 6	0.94	16	6
A28	10 × 7	1.17	−7	−15
